# Ultrasound-Guided Botulinum Neurotoxin Injection for Alleviating Cricopharyngeus Muscle Spasticity: A Cadaveric Feasibility Study with Nerve Ending Analysis

**DOI:** 10.3390/toxins16070317

**Published:** 2024-07-12

**Authors:** Ji-Hyun Lee, Hyung-Jin Lee, Bo Hae Kim

**Affiliations:** 1 Department of Anatomy and Acupoint, College of Korean Medicine, Gachon University, 1332, Seongnam-daero, Seongnam 13120, Republic of Korea; anatomy@gachon.ac.kr; 2 Department of Anatomy, CHA University School of Medicine, 335, Pangyo-ro, Seongnam 13448, Republic of Korea; 3 Department of Otorhinolaryngology-Head and Neck Surgery, Dongguk University Ilsan Hospital, College of Medicine, 27 Dongguk-ro, Goyang 10326, Republic of Korea

**Keywords:** botulinum toxins, deglutition disorders, muscle spasticity, ultrasonography, upper esophageal sphincter

## Abstract

Botulinum neurotoxin (BNT) injection into the cricopharyngeus muscle (CPM) under ultrasound (US) guidance is a minimally invasive technique performed to relieve cricopharyngeal dysphagia by reducing CPM spasticity. This technique is basically accessible only to both lateral sides of the CPM. This cadaveric study aimed to evaluate whether US-guided injection could effectively deliver BNT to abundant areas of gross nerve endings within the CPM. We utilized a newly modified Sihler’s staining method to identify regions with abundant neural endings within the CPM while preserving the three-dimensional morphology of the muscle in 10 sides of 5 fresh cadavers. A mixture of 0.2 mL dye was injected into the 16 sides of CPM under US guidance in 8 cadavers. Nerve endings were abundant in posterolateral areas of the CPM; the injected dye was identified at the posterolateral area on 12 sides (12/16 side, 75%) without diffusion into the posterior cricoarytenoid muscle. The injection failed on four sides (two sides of the prevertebral fascia and two sides of the esophagus below the CPM). These results suggest that US-guided injection could be a feasible technique as it can deliver BNT to the most abundant nerve distribution areas within the CPM in most cases.

## 1. Introduction

The spasticity of the upper esophageal sphincter due to various etiologies, including stroke, brain tumor, or head and neck diseases, can result in transition failure of a bolus from the pharynx to the esophagus, potentially leading to aspiration [1]. Since the cricopharyngeus muscle (CPM) mainly contributes to the pressure generation of the upper esophageal sphincter, impairment of upper esophageal sphincter relaxation is called “cricopharyngeal dysphagia (CPD)” [2,3]. CPD causes malnutrition and aspiration pneumonia, which impairs the quality of life and increases the risk of mortality [4]. Therefore, various interventions for improving CPD have been introduced [3]. Among the interventions for alleviating CPM spasticity, botulinum neurotoxin (BNT) injection is a minimally invasive treatment that can relax the CPM by reducing acetylcholine release at the neuromuscular junctions [5].

BNT injection into the CPM has been conventionally conducted using suspension laryngoscopy or endoscopic assistance under general anesthesia or sedation [6,7]. However, owing to the invasiveness and cumbersomeness of these procedures, a transcutaneous approach for delivering BNT into the CPM has been proposed as an alternative procedure that can be performed in an outpatient setting without general or topical anesthesia [8,9]. In addition, the transcutaneous (transcervical) approach is advantageous in terms of invasiveness and simplicity compared to conventional techniques. Therefore, transcutaneous BNT injection has been recently recommended as a feasible initial option for delivering BNT into the CPM, if available [9,10]. 

For effective relaxation of targeted muscle by BNT injection with minimal complications, accurate drug delivery into the muscle, especially the areas with the most abundant neuromuscular junctions, is necessary [11,12]. Since the CPM has a very small volume, ancillary guidance techniques, including electromyogram (EMG) or ultrasonography (US), have been used for precise BNT delivery [8,11,13]. The successful placement of the injection needle within the CPM is determined by identifying the high-frequency tonic EMG activity at rest, the EMG signal loss during swallowing, and the rebound recruitment pattern after the completion of swallowing [4,8,11]. 

The CPM is mainly innervated by pharyngeal plexus (PP) and recurrent laryngeal nerve (RLN) [2]. The posterolateral area of CPM is known to be the abundant area of neuromuscular junctions [12]. Therefore, previous studies reporting a transcutaneous approach under the EMG guidance have tried to deliver BNT to the lateral and posterolateral areas of the CPM by rotating the larynx [8]. Although the US-guided BNT injection technique is basically accessible only to both lateral sides of the CPM, the clinical improvement of CPD has been reported even in cases with BNT injected into only one lateral area of the CPM [8,14]. 

The injected BNT is usually distributed within the muscle parallel to the muscle fiber and is well spread through the muscle fascia [15,16]. Therefore, we postulated that BNT injected within lateral CPM can reach the posterolateral area of the CPM, which is the most abundant area of the neuromuscular junction, by spreading. This cadaveric study aimed to confirm the area with the most abundant neural distribution within the CPM using a newly modified Siher’s staining method, which can stain nerves while maintaining the three-dimensional (3D) morphology of the CPM. Based on this result, we evaluated the technical feasibility of US-guided BNT injection into the CPM and whether lateral injection could effectively deliver BNT to abundant innervated areas within the CPM.

## 2. Results

### 2.1. Neural Distribution within the CPM

**Figure 1:**
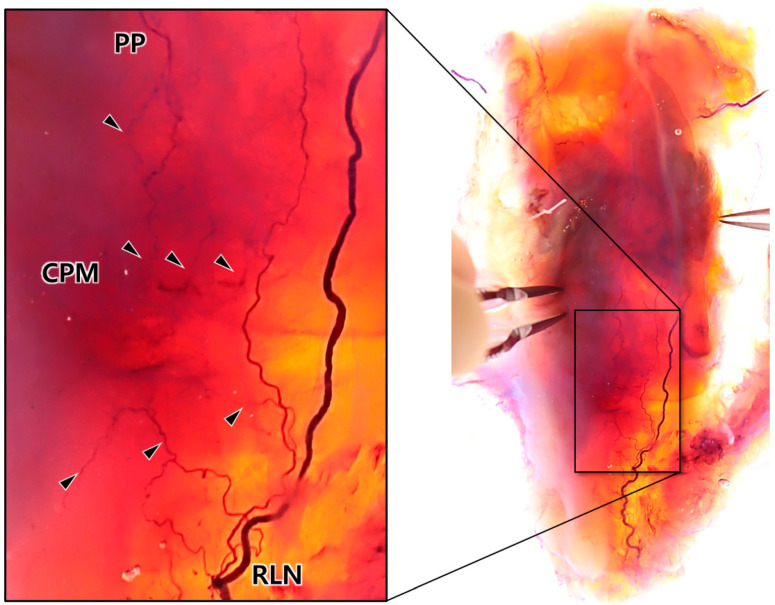
Intramuscular neural distribution of the cricopharyngeus muscle (CPM) as revealed through cartilage-included three-dimensional Sihler’s staining. The innervation patterns within the larynx, including the pharyngeal plexus (PP) of the vagus nerve and the recurrent laryngeal nerve (RLN), are shown. The black arrowheads indicate the small nerve branches distributed within the pharyngeal muscles.

**Figure 2:**
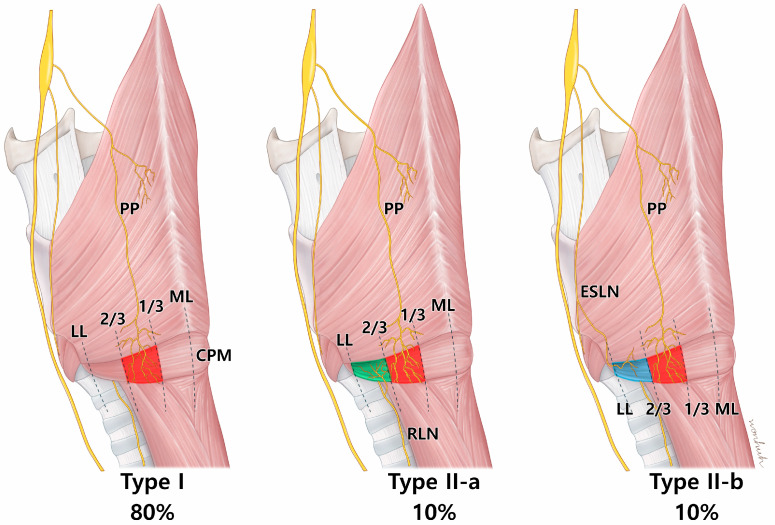
An illustration showing the nerve innervation aspect of the cricopharyngeus muscle (CPM). Type I is characterized by exclusive innervation of the pharyngeal plexus (PP) of the vagus nerve. Type II is characterized by a simultaneous distribution of the PP and branches of the recurrent laryngeal nerve (RLN) or external branch of the superior laryngeal nerve (ESLN). In both patterns, the nerves are mainly localized to the posterolateral side of the muscle. ML; midline, LL; lateral line.

The inclusion of laryngeal cartilages in the newly modified Sihler’s staining process allowed full visualization of the 3D innervation pattern of the larynx (Figure 1). The vagus nerve innervated the CPM, and three distinct patterns of innervation to the CPM were observed (Figure 2, Table 1). The first pattern (Type I) involved the PP of the vagus nerve alone, which was distributed to the posterolateral side of the CPM on eight of 10 sides. The second pattern (Type II-a) included contributions from the RLN to the lateral side, which was observed in one case. The third pattern (Type II-b) involved the external branch of the superior laryngeal nerve (EBSLN) contributing to the lateral side, which was also observed in one case. No gross innervation was observed near or at the midline of the CPM, confirming that, in all cases, the nerves were distributed to the posterolateral side of the CPM.

### 2.2. Evaluation of the US-Guided Injection

**Figure 3:**
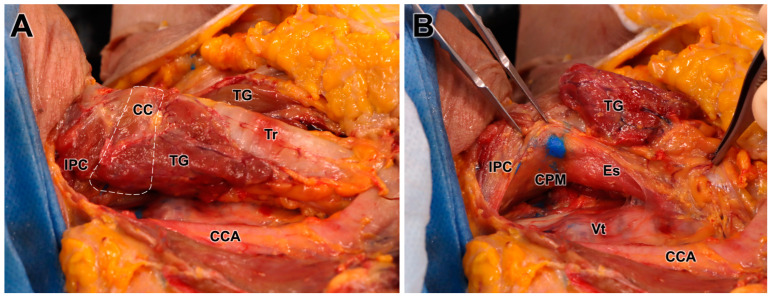
Cadaveric images of the larynx following an ultrasound-guided injection targeting the cricopharyngeus muscle (CPM). Image (**A**) shows the larynx fully exposed, while image (**B**) displays the larynx with the thyroid gland (TG), pharynx, and esophagus (Es) retracted to highlight the area where the blue dye was injected into the posterolateral aspect of the CPM. The dye placement within the CPM is clearly visible. The white dotted line in the image (**A**) indicates the boundary of the cricoid cartilage. CC; cricoid cartilage, IPC; inferior pharyngeal constrictor muscle, TG; thyroid gland (sagittal cut), Tr; trachea, CCA; common carotid artery, Vt; vertebrae.

**Figure 4:**
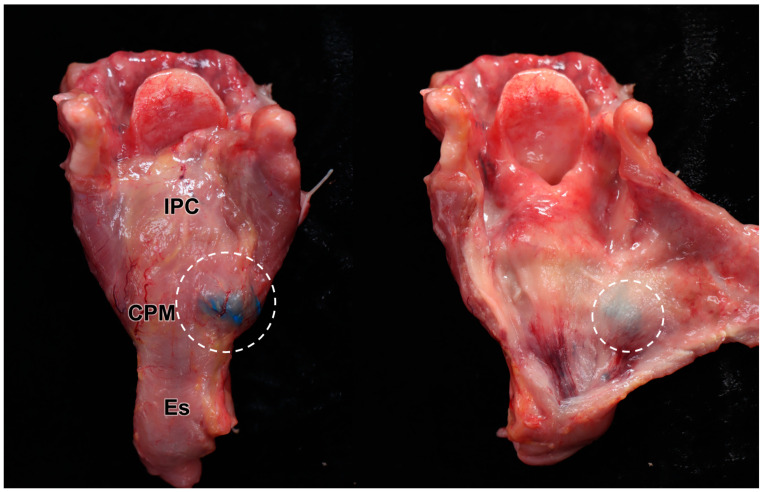
Results of the US-guided injection: The larynx was detached from the cadaver, and the connective tissue was removed, exposing the blue dye localized solely on the posterolateral side of the cricopharyngeus muscle (CPM) (left image). The dye remained strictly confined within the CPM, with no diffusion into adjacent laryngeal structures (right image). The specimen’s darker appearance is due to formalin fixation and preservation. The white circles indicate the injected dye. IPC; inferior pharyngeal constrictor muscle, Es; esophagus.

Of the 16 dye injection trials, 12 injections were accurately placed in the targeted CPM (12/16, 75%) (Figure 3). The success rate was not statistically different between the right (5/8, 62.5%) and left (7/8, 87.5%) sides on Fisher’s exact test (*p* = 0.569). In the four cases where injection into the CPM failed, two cases were identified in the esophagus, one in the prevertebral fascia, and one in the thyroid gland (Table 2). We recorded no case of injected dye diffusing into the inferior constrictor muscle or posterior cricoarytenoid muscle (Figure 4).

## 3. Discussion

In this study, we investigated the CPM nerve innervation and the feasibility of BNT injections for CPD under US guidance. The CPM was innervated by PP and RLN, which are mainly arborized within the posterolateral region on both sides of the CPM. Therefore, BNT delivery to the posterolateral area of the CPM is considered a crucial point for effectively relieving CPD regardless of the left or right side. The dye injected into the lateral area of the CPM under US guidance reached the posterolateral area, the most abundant area in the neural distribution in 75% of cases.

Precisely delivering BNT to the abundant nerve endings within the target muscle facilitates effectively alleviating spasticity with a low dose and volume of BNT, which can minimize unexpected complications and BNT resistance associated with diffusion [16,17]. Therefore, nerve-ending analysis, used to determine the target area of BNT injection in muscle, provides useful information for the effective and safe use of BNT [18]. We have previously investigated the neural distribution within the CPM using Sihler’s staining technique [12]. Conventional Sihler’s staining requires the removal of structures with high calcium content, such as tracheal and laryngeal cartilages, to stain intramuscular nerves [19,20]. However, detaching the CPM from the laryngotracheal framework does not clearly maintain the 3D morphology of the CPM [12]. Therefore, we modified the conventional Sihler’s staining technique to include cartilaginous structures and preserve the 3D morphology of the CPM, allowing the laryngeal framework and CPM to be stained simultaneously. Based on the anatomical review using 3D Sihler’s staining, we found that the most abundant area in neural distribution originated from the PP of the vagus nerve, RLN, and EBSLN, and was located in the posterolateral areas of the CPM. These findings were consistent with previous studies [12]. Therefore, BNT delivery at the posterolateral area of the CPM was thought to be the most important point for the effective injection of BNT into the CPM.

Specifically, 3D staining of the larynx allowed for a more precise and comprehensive analysis and understanding of the innervation within the CPM in this study. This transition provided several significant advantages in neural distribution analyses of the laryngotracheal structures. First, accurate localization and analysis were possible. The 3D approach facilitated a detailed examination of how nerves interconnect within the muscles and cartilages (e.g., the laryngotracheal framework) in their natural anatomical positions. This enabled insights that were not possible with two-dimensional (2D) views or traditional Sihler’s staining. Second, the 3D staining potentially reduced the specimen’s structural distortion compared to traditional 2D Sihler’s staining methods [19]. Unlike 2D techniques that may require the detachment of muscles, as in our previous study, this approach likely preserved the anatomical integrity of the structures more effectively, thereby minimizing alterations in size or shape and enhancing the reliability of the data and the accuracy of nerve distribution depictions. Third, the 3D data improved the clinical applicability of the findings. By maintaining anatomical accuracy, the 3D information offered insights that were directly applicable in clinical settings, supporting more reliable clinical decision making. 

Transcutaneous BNT injection into the CPM is gaining attention due to its demonstrated improvement in CPD despite its simplicity and minimal invasiveness [8,10]. However, CPM has structures around it that can cause major bleeding during and after injection, such as the thyroid gland and carotid artery. In addition, the volume of the CPM is very small [13]. Therefore, a delicate injection technique is required, and can be achieved through ancillary guidance techniques such as EMG or US [10,11]. Although the US-guided technique for BNT injection relies solely on visual feedback to determine whether the needle is accurately inserted into the CPM, the high accessibility of clinicians to US drives its application for transcutaneous BNT injection [11]. The transcutaneous approach makes it relatively easy to access the lateral area of the CPM because the CPM is attached to the lateral side of the cricoid cartilage [4,9]. However, the posterolateral area of the CPM with the most abundant neural distribution is located in a deep position, making direct observation of the posterolateral area of the CPM itself difficult because of the limited transmission potential of US [21]. In addition, manual rotation of the larynx, which increases the accessibility to the posterolateral area of the CPM, is also difficult because the US probe must be manipulated with one hand while injecting with the other during US-guided BNT injection. This technical limitation of US-guided BNT injection into the CPM (i.e., the difficulty of direct access to the posterolateral area of the CPM) should be addressed to improve the effectiveness of US-guided BNT injection. 

The initial step for precise US-guided BNT injection into the CPM is identifying the CPM within the US view [21]. Since the left lateral areas of the CPM attached to the cricoid cartilage are superficially located close to the US probe, this area was relatively well visualized in this study. Therefore, the lateral areas of the CPM have become the optimal target point for BNT injection when using the US-guided technique. However, the CPM attached to the right side of the cricoid cartilage is not well visualized compared to that on the left side [11,21,22]. This point is thought to account for the injection failure rate being somewhat high on the right side, even though there was no statistical difference in success rate between both sides. Therefore, the cricoid cartilage is the most important landmark for advancing the injection needle into the CPM, especially in the right-side procedure. In the present study, the cricoid cartilage was used as a reliable landmark for US-guided injections to locate the CPM [8,9]. We recommend scanning the longitudinal and transverse planes to identify detailed anatomical structures surrounding the larynx, including the CPM, prior to localization. The first step is to identify the cricoid cartilage by palpation, followed by scanning the neck in the longitudinal plane to establish the transverse position of the US probe. When positioning the US probe transversely, the sternocleidomastoid muscle, thyroid gland, inferior border of the cricoid cartilage, common carotid artery, and the surface of the vertebral body served as useful landmarks to identify the CPM before injection [21]. After localizing the CPM via the US, we recommend advancing the needle along the surface of the cricoid cartilage, considering its proximal relationship with the CPM [8,9]. Utmost care should be taken during needle insertion to ensure that the thyroid gland and common carotid artery are not damaged along the needle’s path and that the needle tip does not project beyond the CPM, potentially reaching the prevertebral space and fascia (e.g., the surface of the vertebral body). As the needle is not advanced once it reaches the prevertebral fascia, it should be pulled back in order to find the needle tip in this situation.

The injected dye into the CPM in this study was observed in both lateral and posterolateral areas in 75% of the sides of the CPM, even though only 0.2 mL of the dye was injected. Therefore, a limited volume of BNT injection into the lateral area of the CPM under US guidance is thought to be sufficient for relaxing the CPM. Two mechanisms may account for the injected dye reaching the posterolateral area of the CPM. Rotating the head to the opposite side to be injected may allow exposure of the posterolateral area. In addition, the spread of injected dye along muscle fiber may be another mechanism [16]. 

The complications of BNT injection, mainly caused by injection at the other structures around the CPM or unexpected spread or diffusion, may come in various forms, including worsening dysphagia or vocal fold paralysis [6,8,9,23]. When considering the anatomical location, the abduction failure of the vocal fold caused by the incorrect injection or spread of BNT into the posterior cricoarytenoid muscles can also occur [12]. There was no case of the injected dye spreading to the intralaryngeal muscles, including the posterior cricoarytenoid muscle [24]. In addition, the injected dye was not found in the inferior constrictor muscle, which can weaken the pharyngeal contraction during swallowing in the presence of BNT [24]. Therefore, US-guided BNT injection with a small volume into the CPM seems to be safe and unlikely to impair the swallowing mechanism, especially in the pharyngeal phase. However, there are two possible complications that can be inferred from the dye found in the thyroid gland in one case. The presence of the dye within the thyroid gland clearly implies that the needle punctured the thyroid gland. Therefore, careful observation is necessary after the procedure to determine the formation of a hematoma [25]. In addition, systemic circulation of BNT can occur, considering the abundance of blood flow in the thyroid gland [26]. The unpredictable complications caused by BNT spread along the esophagus and prevertebral fascia also can occur [26]. Therefore, counseling regarding unexpected and elusive systemic complications, including systemic muscle weakness caused by US-guided BNT injection into the CPM, should be provided to patients before the procedure. 

This study has some limitations that should be acknowledged. First, since the study was conducted on cadavers, we were unable to compare the EMG and US-guided injection techniques or validate the simultaneous use of these guidance methods. Second, the number of cadavers used for Sihler’s staining was small, limiting the statistical significance of the results. Third, the cadavers used in this study were all from a single race and were of older adults. Fourth, the spread of BNT within the CPM of a living human due to gravity and muscle contraction cannot be clearly replicated even in fresh cadavers.

## 4. Conclusions

The posterolateral area of the CPM is the most abundant area of nerve endings. Since the US-guided injection can deliver the BNT to the posterolateral area of the CPM without manual rotation in most cases, this technique seems to be a feasible option for effectively alleviating CPM spasticity. However, BNT can be injected into unexpected areas, so careful monitoring of complications after the BNT injection is necessary.

## 5. Materials and Methods

### 5.1. Cadaver Dissection for Specimen Collection

This cadaveric study was conducted in compliance with the Act on Dissection and Preservation of Corpses of the Republic of Korea (Act number: 14885) and was approved by the Institutional Review Board of our institution (Approval No. MC22SISI0098). All donors or authorized representatives provided written informed consent for the use of the cadavers and consent for their use in future research on the related materials.

A total of 13 fresh cadavers were utilized for this study, including 5 cadavers (3 males and 2 females; median age, 77.7 years (range: 63.7–99.1)) for which the 10 CPM sides were employed for identifying the intramuscular neural distribution and its endings within the CPM; and 8 cadavers (5 males and 3 females; median age, 76.5 years (range: 49.6–90.3)) for which the 16 CPM sides were used to investigate the feasibility of US-guided BNT injection into the CPM. None of the cadavers included in this study had experienced trauma, surgery, or deformity in the head and neck regions, including the larynx.

Dissection was conducted by carefully removing the skin, subcutaneous tissue, suprahyoid and infrahyoid muscles, the thyroid gland, and the blood vessels to explore the intramuscular neural distribution and its nerve endings within the CPM. This process exposed the following anatomical structures: the main laryngeal cartilages, including the thyroid and cricoid cartilages, laryngeal muscles, the internal branch of the superior laryngeal nerve, and the RLN.

### 5.2. Newly Modified Sihler’s Staining and Evaluation of Neural Distribution

Sihler’s whole-mount nerve staining was used to examine the intramuscular neural distribution within the CPM [12,27]. We modified the previously reported staining method to stain the entire larynx, including the thyroid cartilage, cricoid cartilage, and laryngeal muscles. The detailed staining process is as follows:

- Fixation: The procured larynx was washed under running tap water and fixed in a 10% neutral formalin solution for 1 week.
- Maceration and depigmentation: The fixed larynx was soaked in 3% aqueous potassium hydroxide solution (0.01% hydrogen peroxide could be added for further depigmentation) for more than 2 weeks to soften and depigment the tissue. The duration was adjusted based on the softening state of the tissue.
- Decalcification and whitening: The softened larynx was washed under running tap water, then transferred to Sihler I Solution (a mixture of glycerin, glacial acetic acid, and distilled water in a ratio of 1:1:6) to remove calcium from the cartilage and whiten the tissue. This process took more than 3 weeks and continued until the cartilage was sufficiently softened.
- Staining: The entire tissue, including muscles and nerves, was stained with Sihler II solution (comprising 10% Ehrlich’s hematoxylin, 10% glycerin, and distilled water) for 3 days. This period could be extended if necessary.
- Destaining: The stained larynx was transferred to Sihler I Solution to remove the stain from tissues other than nerves. This process was monitored closely and typically lasted for several hours (usually 3–6 h). The solution was replaced as needed until the staining of non-neural tissues was adequately removed.
- Neutralization and blueing: The tissue was soaked under running tap water for 3–6 h to neutralize the tissue and enhance the staining of the nerves.
- Dehydration: After staining, the larynx was dehydrated using a graded series of ethanol solutions (70–99.9%).
- Clearing: The dehydrated larynx was placed in 99% methyl salicylate to render the tissue transparent.

The processed tissues were then observed on a light view box. Each side of the muscle was divided into three parts using vertical lines dividing the width into thirds to analyze the innervation pattern within the CPM, which resulted in an even division of each side of the CPM into lateral, posterolateral, and posterior areas. The pattern of intramuscular distribution and the accuracy of injection were then analyzed based on these areas.

### 5.3. US Scanning Protocol

Utilizing the HS50 device (Samsung, Seoul, Republic of Korea) coupled with a linear array transducer (LA3-14AD, 3–14 MHz), we performed real-time B-mode US scanning. Before scanning, the head of the cadaver was rotated as far as possible to the opposite side to ensure that the skin of the laryngeal area and the US transducer were perpendicular, facilitating easy US imaging. Then, the anterior side of the neck was palpated to identify and mark the cricoid cartilage, which is a key anatomical landmark of the larynx.

A single experienced head and neck surgeon who boasts over a decade of expertise in head and neck imaging and BNT injection into the larynx conducted the procedures.

The neck of the cadavers was extended as much as possible to facilitate application of the probe at an approximately 90° angle to the skin. By palpating the anterior side of the neck, we identified the thyroid and cricoid cartilages, which are key anatomical landmarks for applying various transcutaneous procedures into the larynx. The superior and inferior boundaries of the cricoid cartilage were marked at the skin. The transducer was aligned transversely to conduct scans across the inferior boundary of the cricoid cartilage guided by demarcations and anatomical features. When the transducer was tilted slightly upward at the lower boundary of the cricoid cartilage, strap muscles, cricoid cartilage, thyroid gland, and carotid artery could be observed simultaneously on US.

### 5.4. US-Guided Injection Procedure and Evaluation of the Injected Dye Distribution

**Figure 5:**
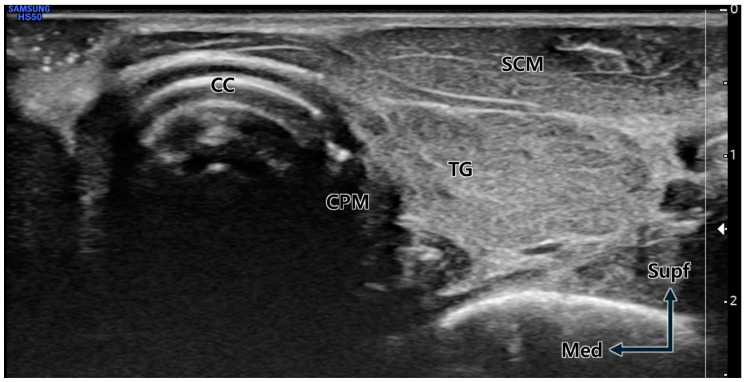
Transverse ultrasound image at the cricoid cartilage (CC) level. Due to the neck rotation to the lateral side, the cricopharyngeus muscle (CPM) is prominently visible immediately lateral (posterior) to the distinctly observed CC. TG; thyroid gland, SCM; sternocleidomastoid muscle, Supf; superficial, Med; medial.

The US-guided injection procedures were conducted using a 6 cm long 24G needle, with the target depth preliminarily determined through US imaging. For observation of the cricoid cartilage, CPM, and common carotid artery, the transducer was placed on the lateral border of the cricoid cartilage using the out-of-plane method (Figure 5).

The needle was inserted percutaneously at the middle level of the cricoid cartilage on its lateral side and then carefully advanced by tapping the cricoid cartilage while verifying the position of the carotid artery to avoid puncture injury [8,9]. Upon accurately placing the needle within the designated muscle, CPM, 0.2 mL of blue or green dye was administered.

Following the US-guided injections, each cadaver was meticulously examined to assess whether the dye accurately reached the CPM and properly spread to the area within the CPM where a significant concentration of nerve endings is located. In addition, we checked whether the dye was injected into unexpected structures, especially the neurovascular structures, esophagus, inferior constrictor muscle, and intralaryngeal muscles. The verification of dye targeting post-injection was conducted through the detailed dissection technique outlined in Section 5.1, titled “Cadaver Dissection for Specimen Collection”.

We defined success as the presence of dye in the CPM and failure as the absence of dye in the CPM. The difference in the success rate of BNT injection between the right and left sides of the CPM was compared using Fisher’s exact test, analyzed with SPSS software version 22.0 (IBM Corp., 2013. IBM SPSS Statistics for Windows, Version 22.0. Armonk (N.Y., USA): IBM Corp.).

## Figures and Tables

**Table 1:** Intramuscular nerve distribution pattern by each side of the larynx.

Specimen	Sides	
	Left	Right
1	PP	PP
2	RLN/PP	PP
3	PP	PP
4	PP/ESLN	PP
5	PP	PP

PP, pharyngeal plexus; RLN, recurrent laryngeal nerve; ESLN, external branch of the superior laryngeal nerve.

**Table 2:** Injection results.

Specimen	Sides	Suitability (Posterolateral Side of the CPM)	Diffusion Area
1	Lt	○	
	Rt	○	
2	Lt	○	
	Rt	○	
3	Lt	○	
	Rt	○	
4	Lt	X	Prevertebral fascia
	Rt	X	Esophagus
5	Lt	○	
	Rt	X	Thyroid
6	Lt	○	
	Rt	X	Esophagus
7	Lt	○	
	Rt	○	
8	Lt	○	
	Rt	○	

CPM, cricopharyngeus muscle; Lt, left; Rt, right; ○, injection accurately placed in the targeted CPM; X, injection into the CPM failed.

## Data Availability

The data presented in this study are available on request from the corresponding author due to privacy and ethical restrictions.
